# Supplementary material for: Adaptive variation in avian eggshell gas conductance and structure across elevational gradients?
Source: eLife. 2026 Apr 14;15:e85564. doi: 10.7554/eLife.85564 (PMC13152275; doi:10.7554/eLife.85564)
Supplement: Supplementary file 4. [file elife-85564-supp4.docx]

Research Article

**Adaptive variation in avian eggshell gas conductance and structure across elevational gradients?**

David Ocampo, Carlos Daniel Cadena, Esteban Correa-Agudelo, Marcela Hernández Hoyos and Gustavo A. Londoño

**Supplementary File 4**. Akaike Information Criterion (AIC) and delta AIC (ΔAIC) values used to compare alternative models of evolution for the eggshell traits in phylogenetic analyses.

| Models | | AIC | Delta AIC |
| --- | --- | --- | --- |
|  |  |  |  |
| Conductance | |  |  |
|  | *Pagel* | 923.04 | 0.00 |
|  | *Brownian Motion* | 981.61 | 62.18 |
|  | *Ornstein-Uhlenbeck (alpha = 1)* | 925.13 | 2.93 |
|  | *Early-Burst* | 983.61 | 64.29 |
|  |  |  |  |
| Shell thickness | |  |  |
|  | *Pagel* | -249.14 | 13.85 |
|  | *Brownian Motion* | -211.95 | 51.04 |
|  | *Ornstein-Uhlenbeck (alpha = 1)* | -262.99 | 0.00 |
|  | *Early-Burst* | -209.85 | 53.14 |
|  |  |  |  |
| Pore density | |  |  |
|  | *Pagel* | 7.26 | 1.00 |
|  | *Brownian Motion* | 30.78 | 24.54 |
|  | *Ornstein-Uhlenbeck (alpha = 1)* | 6.25 | 0.00 |
|  | *Early-Burst* | 32.88 | 26.63 |
|  |  |  |  |
| Pore size | |  |  |
|  | *Pagel* | 947.62 | 7.85 |
|  | *Brownian Motion* | 974.90 | 35.11 |
|  | *Ornstein-Uhlenbeck (alpha = 1)* | 939.79 | 0.00 |
|  | *Early-Burst* | 977.02 | 37.23 |

**References for supplementary tables**

Remsen, J. V., Jr., C. D. Cadena, A. Jaramillo, M. Nores, J. F. Pacheco, J. Pérez-Emán, M. B. Robbins, F. G. Stiles, D. F. Stotz, and K. J. Zimmer. 2020. A classification of the bird species of South America. American Ornithologists' Union. http://www.museum.lsu.edu/~Remsen/SACCBaseline.html
